# Supplementary material for: Household food insecurity and early childhood development: Longitudinal evidence from Ghana
Source: PLoS One. 2020 Apr 3;15(4):e0230965. doi: 10.1371/journal.pone.0230965 (PMC7122750; doi:10.1371/journal.pone.0230965)
Supplement: S1 Material — (DOCX) [file pone.0230965.s001.docx]

**S1 File. Differences in child and household characteristics in wave 1 by attrition status.**

|  | Included  (*n* = 1,333) | Excluded  (*n* = 875) | *F*- or χ^2^ statistic | p-value |
| --- | --- | --- | --- | --- |
| Literacy (0-1) | 0.467 | 0.466 | 0.01 | 0.911 |
| Numeracy (0-1) | 0.449 | 0.445 | 0.27 | 0.604 |
| Social-emotional (0-1) | 0.416 | 0.412 | 0.20 | 0.651 |
| Executive function (0-1) | 0.497 | 0.481 | 2.88 | 0.090 |
| Approaches to learning (0-4) | 3.09 | 3.07 | 0.61 | 0.435 |
| District |  |  | 2.92 | 0.712 |
| District 1 | 15.1% | 14.7% |  |  |
| District 2 | 25.2% | 23.3% |  |  |
| District 3 | 14.0% | 16.5% |  |  |
| District 4 | 23.3% | 22.9% |  |  |
| District 5 | 13.2% | 13.1% |  |  |
| District 6 | 9.2% | 9.5% |  |  |
| Sex male | 50.4% | 49.4% | 0.28 | 0.63 |
| Grade level |  |  | 2.46 | 0.29 |
| Kindergarten 1 | 46.5% | 47.9% |  |  |
| Kindergarten 2 | 43.5% | 44.0% |  |  |
| Combined class | 10.1% | 8.1% |  |  |
| Age (years) | 5.6 | 5.9 | 28.9 | 0.001 |
| Enrolled in private school | 59.2% | 51.1% | 13.97 | 0.001 |
