# Supplementary material for: Household food insecurity and early childhood development: Longitudinal evidence from Ghana
Source: PLoS One. 2020 Apr 3;15(4):e0230965. doi: 10.1371/journal.pone.0230965 (PMC7122750; doi:10.1371/journal.pone.0230965)
Supplement: S2 Material — (DOCX) [file pone.0230965.s002.docx]

**S2 File. Measurement of wave 1 outcomes.**

As described in section 2.2.1, assessments for literacy and numeracy were different at wave 1 than in wave 3 due to growth in these skills areas over the three years of the study. Below, we describe the measures used at Wave 1. In all analyses, the z-score for each respective outcome is included in the analysis. The instrument used was the International Development and Early Learning Assessment (IDELA), developed by Save the Children (Pisani et al., 2018). The tool was translated into three local languages (Twi, Ewe, and Ga). Surveys were translated and then back-translated by a different person to check for accuracy. Any discrepancies were discussed and addressed. Finally, after being trained on the instrument, a group of surveyors read and discussed the translated version in their respective local language and made additional changes as a group.

*Early literacy.* The domain of early literacy consisted of 38 items grouped into six subtasks, and it covers constructs of print awareness, letter knowledge, phonological awareness, oral comprehension, emergent writing, and expressive vocabulary. An example subtask on phonological awareness asked children to identify words that begin with the same sound. A sample item is: “Here is my friend mouse. Mouse starts with /m/. What other word starts with /m/? Cow, doll, milk” (*α =* .74).

*Early numeracy.* The domain of early numeracy consisted of 39 items grouped into eight subtasks and covers constructs of number knowledge, basic addition and subtraction, one-to-one correspondence, shape identification, sorting abilities based on color and shape, size and length differentiation, and completion of a simple puzzle. An example item for shape identification showed the child a picture with six shapes and asked the child to identify the circle (*α* = .72).

*Approaches to Learning.* Similar to the measure on self-regulation, approaches to learning was assessor-reported and conducted after completion of the child assessment. After the assessor completed the IDELA items with each child, they filled out seven items about the child’s behaviors during the assessment period. Each child was rated on a scale of 1 to 4, with 1 = “almost never” and 4 = “almost always”. Assessors reported on children’s attention (i.e., “Did the child pay attention to the instructions and demonstrations through the assessment?”), confidence, concentration, diligence, pleasure, motivation, and curiosity during the tasks (*α* = 0.94).
