# Supplementary material for: Household food insecurity and early childhood development: Longitudinal evidence from Ghana
Source: PLoS One. 2020 Apr 3;15(4):e0230965. doi: 10.1371/journal.pone.0230965 (PMC7122750; doi:10.1371/journal.pone.0230965)
Supplement: S3 Material — (DOCX) [file pone.0230965.s003.docx]

**S3 File. Descriptive statistics of covariates, full sample and by household food insecurity status.**

|  | **Full sample** | | | **Never food insecure** | | **Transitory food insecurity** | | **Persistent food insecurity** | | ***F*- or *X*^2^ statistic** | **p-value** |
| --- | --- | --- | --- | --- | --- | --- | --- | --- | --- | --- | --- |
|  | M / % | SD | M / % | | SD | M / % | SD | M / % | SD |  |  |
|  | **Full sample** | | | **Never food insecurity** | | **Transitory food insecure** | | **Persistent food insecure** | |  |  |
| Child male | 0.50 | 0.50 | 0.49 | | 0.50 | 0.53 | 0.50 | 0.55 | 0.50 | 1.36 | 0.505 |
| Child age | 7.68 | 1.29 | 7.63 | | 1.26 | 7.92 | 1.30 | 8.11 | 1.77 | 6.55 | 0.002 |
| Caregiver female | 60.3% |  | 59.4% | |  | 65.9% |  | 63.6% |  | 2.89 | 0.236 |
| Caregiver age | 39.96 | 8.63 | 40.25 | | 8.71 | 38.35 | 8.00 | 38.36 | 8.21 | 4.19 | 0.015 |
| Household size | 5.24 | 1.83 | 5.18 | | 1.87 | 5.58 | 1.51 | 5.59 | 1.40 | 4.43 | 0.012 |
| Private school | 56.8% |  | 59.1% | |  | 44.5% |  | 34.1% |  | 22.80 | 0.000 |
| *Language of assessment* |  |  |  | |  |  |  |  |  |  |  |
| English | 45.9% |  | 46.9% | |  | 42.2% |  | 29.5% |  | 6.21 | 0.045 |
| Twi / Fanti | 19.1% |  | 18.7% | |  | 20.2% |  | 27.3% |  | 2.21 | 0.331 |
| Other | 3.9% |  | 3.4% | |  | 6.4% |  | 9.1% |  | 6.92 | 0.031 |
| Mixed | 31.2% |  | 31.1% | |  | 31.2% |  | 34.1% |  | 0.18 | 0.914 |
| *Treatment status* |  |  |  | |  |  |  |  |  |  |  |
| Treatment status 1 TT | 36.8% |  | 37.5% | |  | 33.5% |  | 27.3% |  | 2.82 | 0.244 |
| Treatment status 2 TTPA | 36.1% |  | 35.6% | |  | 39.3% |  | 38.6% |  | 1.03 | 0.597 |
| Treatment status 3 Control | 27.1% |  | 26.9% | |  | 27.2% |  | 34.1% |  | 1.13 | 0.569 |
| *Caregiver education* |  |  |  | |  |  |  |  |  |  |  |
| No education or some primary | 19.5% |  | 17.9% | |  | 28.8% |  | 26.2% |  | 12.11 | 0.002 |
| Primary or junior secondary | 49.9% |  | 50.3% | |  | 46.6% |  | 52.4% |  | 0.88 | 0.645 |
| Secondary & higher | 30.6% |  | 31.8% | |  | 24.5% |  | 21.4% |  | 5.25 | 0.073 |
| Household asset | 0.05 | 0.98 | 0.13 | | 0.95 | 0.43 | 1.04 | 0.49 | 1.02 | 33.08 | 0.000 |
| School quality | 8.40 | 1.54 | 8.38 | | 1.54 | 8.65 | 1.49 | 8.16 | 1.58 | 2.65 | 0.071 |
| Sample size | 1,333 | | 1,116 | | | 173 | | 44 | |  | |
