# Supplementary material for: Household food insecurity and early childhood development: Longitudinal evidence from Ghana
Source: PLoS One. 2020 Apr 3;15(4):e0230965. doi: 10.1371/journal.pone.0230965 (PMC7122750; doi:10.1371/journal.pone.0230965)
Supplement: S4 Material — (DOCX) [file pone.0230965.s004.docx]

**S4 File. Adjusted value-added models with interaction between household food insecurity and gender.**

|  | Literacy | | Numeracy | | Short-term memory | | Social-emotional | Self-regulation |
| --- | --- | --- | --- | --- | --- | --- | --- | --- |
|  | **Panel A: Any food insecurity** | | | | | | | |
| Reference: Never food insecure | |  | |  | |  | |  |
|  |  |  | |  | |  | |  |
| Any food insecurity | -0.110 | | -0.154* | | -0.176* | | 0.069 | 0.022 |
|  | (-0.300 - 0.080) | | (-0.320 - 0.013) | | (-0.369 - 0.017) | | (-0.176 - 0.314) | (-0.190 - 0.234) |
| Boy | -0.034 | | -0.017 | | 0.023 | | -0.075 | -0.044 |
|  | (-0.131 - 0.064) | | (-0.108 - 0.073) | | (-0.108 - 0.154) | | (-0.204 - 0.054) | (-0.168 - 0.080) |
| Any food insecurity X Boy | -0.009 | | 0.001 | | -0.105 | | -0.060 | -0.270 |
|  | (-0.271 - 0.254) | | (-0.221 - 0.224) | | (-0.406 - 0.197) | | (-0.388 - 0.269) | (-0.614 - 0.073) |
|  | **Panel B: Household food insecurity trajectories** | | | | | | | |
| Reference: Never food insecure | |  | |  | |  | |  |
|  |  |  | |  | |  | |  |
| Transitory food insecurity | -0.037 | | -0.132 | | -0.150 | | 0.068 | -0.032 |
|  | (-0.235, 0.162) | | (-0.313, 0.050) | | (-0.374, 0.073) | | (-0.161, 0.298) | (-0.245, 0.180) |
| Persistent food insecurity | -0.388* | | -0.237 | | -0.273 | | 0.072 | 0.237 |
|  | (-0.797, 0.020) | | (-0.542, 0.067) | | (-0.701, 0.156) | | (-0.554, 0.698) | (-0.230, 0.705) |
| Boy | -0.034 | | -0.017 | | 0.023 | | -0.075 | -0.044 |
|  | (-0.131, 0.063) | | (-0.108, 0.073) | | (-0.108, 0.154) | | (-0.204, 0.054) | (-0.168, 0.080) |
| Transitory food insecurity X Boy | -0.084 | | -0.084 | | -0.164 | | -0.090 | -0.232 |
|  | (-0.356, 0.189) | | (-0.333, 0.165) | | (-0.491, 0.164) | | (-0.441, 0.261) | (-0.593, 0.129) |
| Persistent food insecurity X Boy | 0.276 | | 0.329* | | 0.122 | | 0.056 | -0.424 |
|  | (-0.283, 0.835) | | (-0.041, 0.698) | | (-0.470, 0.713) | | (-0.654, 0.767) | (-1.081, 0.233) |
|  |  | |  | |  | |  |  |
| Observations | 1,261 | 1,261 | | 1,261 | | 1,261 | | 1,232 |
| R-squared | 0.345 | 0.414 | | 0.099 | | 0.053 | | 0.052 |

* p<0.1. Robust confidence intervals in parentheses, standard errors clustered at baseline school-level. Estimates also control for: wave 1 values of the specific outcomes, with the exception of behavioural regulation, for which we control for wave 1 approaches to learning; child gender and age in years; caregiver’s gender, age and education level; treatment arm; household size; language of test administration.
