# Supplementary material for: Household food insecurity and early childhood development: Longitudinal evidence from Ghana
Source: PLoS One. 2020 Apr 3;15(4):e0230965. doi: 10.1371/journal.pone.0230965 (PMC7122750; doi:10.1371/journal.pone.0230965)
Supplement: S5 Material — (DOCX) [file pone.0230965.s005.docx]

**S5 File. Extended adjusted value-added model.**

|  | Literacy | | Numeracy | | | Short-term  memory | | Social-emotional | | | Self-regulation | |
| --- | --- | --- | --- | --- | --- | --- | --- | --- | --- | --- | --- | --- |
|  | Panel A: Household assets | | | | | | | | | | | |
| Reference: Never food insecure | | |  | | |  | |  | | |  | |
| Transitory food insecurity | -0.015 | -0.081 | | | -0.098 | | | | 0.020 | | | -0.050 |
|  | (-0.133, 0.102) | (-0.186, 0.024) | | | (-0.231, 0.036) | | | | (-0.113, 0.153) | | | (-0.195, 0.095) |
| Persistent food insecurity | -0.132* | 0.043 | | | -0.247*** | | | | 0.071 | | | 0.089 |
|  | (-0.276, 0.011) | (-0.099, 0.184) | | | (-0.405, -0.090) | | | | (-0.104, 0.246) | | | (-0.092, 0.270) |
| Asset index | 0.121*** | 0.114*** | | | 0.061* | | | | 0.075** | | | 0.152*** |
|  | (0.067, 0.175) | (0.061, 0.166) | | | (-0.004, 0.126) | | | | (0.001, 0.150) | | | (0.087, 0.217) |
| Observations | 1,258 | 1,258 | | | 1,258 | | | | 1,258 | | | 1,229 |
| R-squared | 0.356 | 0.421 | | | 0.102 | | | | 0.057 | | | 0.066 |
|  | Panel B: School quality | | | | | | | | | | | |
| Reference: Never food insecure | | | |  | | |  | | |  | | |
| Transitory food insecurity | -0.038 | -0.117** | | | -0.090 | | | | 0.015 | | | -0.055 |
|  | (-0.160, 0.085) | (-0.231, -0.002) | | | (-0.230, 0.049) | | | | (-0.127, 0.157) | | | (-0.215, 0.105) |
| Persistent food insecurity | -0.239*** | -0.070 | | | -0.327*** | | | | -0.002 | | | -0.022 |
|  | (-0.397, -0.081) | (-0.218, 0.078) | | | (-0.487, -0.167) | | | | (-0.194, 0.190) | | | (-0.227, 0.183) |
| School quality | 0.083** | 0.009 | | | -0.023 | | | | 0.005 | | | 0.036 |
|  | (0.020, 0.145) | (-0.046, 0.063) | | | (-0.103, 0.057) | | | | (-0.081, 0.090) | | | (-0.021, 0.093) |
| Observations | 1,104 | 1,104 | | | 1,104 | | | | 1,104 | | | 1,076 |
| R-squared | 0.343 | 0.407 | | | 0.111 | | | | 0.043 | | | 0.043 |
|  | Panel C: Private school | | | | | | | | | | | |
| Reference: Never food insecure | | | |  | | |  | | |  | | |
| Transitory food insecurity | -0.040 | -0.086 | | | -0.104 | | | | 0.000 | | | -0.058 |
|  | (-0.157, 0.077) | (-0.189, 0.017) | | | (-0.233, 0.025) | | | | (-0.132, 0.133) | | | (-0.201, 0.085) |
| Persistent food insecurity | -0.181** | 0.036 | | | -0.263*** | | | | 0.036 | | | 0.072 |
|  | (-0.324, -0.038) | (-0.094, 0.165) | | | (-0.413, -0.113) | | | | (-0.139, 0.211) | | | (-0.100, 0.244) |
| Private school | 0.155** | 0.366*** | | | 0.119 | | | | 0.079 | | | 0.408*** |
|  | (0.023, 0.287) | (0.262, 0.469) | | | (-0.037, 0.275) | | | | (-0.087, 0.245) | | | (0.254, 0.562) |
| Observations | 1,261 | 1,261 | | | 1,261 | | | | 1,261 | | | 1,232 |
| R-squared | 0.350 | 0.439 | | | 0.102 | | | | 0.053 | | | 0.084 |
|  | Panel D: Household assets, school quality, and private school | | | | | | | | | | | |
| Reference: Never food insecure | | | |  | | |  | | |  | | |
| Transitory food insecurity | -0.009 | -0.103 | | | -0.164** | | | | 0.101 | | | -0.010 |
|  | (-0.160, 0.141) | (-0.247, 0.040) | | | (-0.317, -0.011) | | | | (-0.079, 0.282) | | | (-0.182, 0.161) |
| Persistent food insecurity | -0.256* | -0.016 | | | -0.262* | | | | 0.081 | | | 0.103 |
|  | (-0.539, 0.028) | (-0.238, 0.205) | | | (-0.571, 0.048) | | | | (-0.380, 0.543) | | | (-0.277, 0.483) |
| Household assets | 0.074*** | 0.043 | | | 0.074** | | | | 0.059 | | | 0.079** |
|  | (0.018, 0.131) | (-0.010, 0.096) | | | (0.007, 0.141) | | | | (-0.018, 0.135) | | | (0.012, 0.147) |
| School quality | 0.082** | 0.020 | | | -0.025 | | | | 0.000 | | | 0.044 |
|  | (0.019, 0.144) | (-0.035, 0.074) | | | (-0.105, 0.054) | | | | (-0.087, 0.087) | | | (-0.013, 0.101) |
| Private school | 0.164** | 0.362*** | | | 0.081 | | | | 0.031 | | | 0.344*** |
|  | (0.024, 0.304) | (0.252, 0.472) | | | (-0.095, 0.257) | | | | (-0.145, 0.207) | | | (0.183, 0.505) |
| Observations | 1,101 | 1,101 | | | 1,101 | | | | 1,101 | | | 1,073 |
| R-squared | 0.353 | 0.441 | | | 0.114 | | | | 0.048 | | | 0.083 |

**** p<0.01, ** p<0.05, * p<0.1. Robust confidence intervals in parentheses, standard errors clustered at baseline school-level. Estimates also control for: wave 1 values of the specific outcomes, with the exception of task orientation, for which we control for wave 1 approaches to learning (as task orientation was not collected at baseline); child gender and age in years; caregiver’s gender, age and education level; treatment arm; household size; language of test administration. The asset index is the predicted score from the first component of a principal component analysis of the durable assets the household owns and provides a proxy for the household´s economic position. The school quality score assesses the nature of teacher-child interactions in low-resourced settings. Both indices have mean of 0 and standard deviation of 1. Private school is a dichotomous variable assuming the value of 1 if the child is enrolled in a private school, 0 if she is enrolled in a government school.*
