# Supplementary material for: Household food insecurity and early childhood development: Longitudinal evidence from Ghana
Source: PLoS One. 2020 Apr 3;15(4):e0230965. doi: 10.1371/journal.pone.0230965 (PMC7122750; doi:10.1371/journal.pone.0230965)
Supplement: Attachment — Submitted filename: Response_to_Reviewers.docx [file pone.0230965.s006.docx]

We greatly appreciate the time that the editor and reviewers took to review our manuscript and provide helpful feedback and comments. We have made a variety of edits in response that we believe address all the issues raised by the reviews. We believe that this feedback and the subsequent revisions have made this manuscript stronger and more compelling. Once again, we are very grateful for the time and attention that went into these reviews. Below, we include a point-by-point reply to each of the comments, including a description of where we made changes to the manuscript (with new/added text shown in *italics*).

**Reviewer #1**

1. The discussion of limitations does well in describing the main limitations, but pays scant attention to the resulting threats to 'conclusion validity'. How might the reported estimates of the relationship between household food insecurity and child development have been affected by the sampling issues (urban sample frame and selection bias)? Also school attendance rates are fairly high in Greater Accra and less so in rural areas of the country. What role might school attendance in itself have in promoting child development? This is a constant in the present study, and only field survey methods could address this question (not school-based research). However, these issues do not seriously detract from the high value of the study, and this report is ready for publication.

**Response:** Thank you for raising these important limitations; the limitations to external validity for the reasons the reviewer raised are critical. We had added them to the limitations section on page 24 stating: “*Our reported estimates may have been affected by sampling issues related to the urban and peri-urban sampling frame and selection bias, as children’s outcomes were measured at school and not at home, thus not capturing out-of-school children, which may be especially vulnerable to food insecurity. Furthermore, school attendance rates are relatively higher in these areas compared to rural areas, which may play an important role in influencing child outcomes*.”

**Reviewer #2**

2. My primary concern is with the authors’ measures of household food insecurity. Specifically, the way the authors have defined food insecurity (lines 248-259) is not consistent with the way the household hunger scale (HHS) was developed and validated for cross-cultural use. There are two primary issues. First, the HHS questions consist not just of occurrence questions but also frequency (i.e., each occurrence question is followed-up with a question asking how frequently in the past 4 weeks the household experienced the particular food insecurity condition). According to the HHS guidelines (<https://www.fantaproject.org/sites/default/files/resources/HHS-Indicator-Guide-Aug2011.pdf>), ALL questions must be asked and incorporated into the indicator of food insecurity (p. 5): “To collect HHS data, it is very important that this full set of HHS questions be used. Project staff should not pick and choose certain HHS questions for inclusion in the questionnaire, because it is the set of HHS questions—not the use of each HHS question independently—that has been validated as a meaningful measure of household food deprivation.” Did the authors collect data on the frequency of occurrence questions? Then, as indicated on page 12 of the HHS guidelines, the response questions should be recoded and, based on the sum of the household score on each question, households are then categorized as having little to no hunger if their score is 0-1, moderate hunger for scores 2-3, and severe hunger for scores 4-6. The way the authors categorized food insecurity (any household that answered yes to any one of the food insecurity occurrence questions without regard to frequency) may very well have categorized households as food insecure when, according to the actual/validated HHS, they should have been categorized as food secure. If the authors did collect the frequency of occurrence questions, then they could redo their categorization by following the HHS guidelines and rerun all of their analyses. If not, I’m afraid their measures of food insecurity are not valid. In lines 495-496 the authors claim that a strength of their analysis is that they’ve used a “cross-context validated measure of food insecurity”, but unless I am completely misunderstanding the way you constructed your food insecurity indicator, or unless there is some alternative, validated method for using the HHS questions of which I am not aware, I’m afraid you have not used a validated measure of food insecurity.

**Response:** We thank the reviewer for bringing this issue to our attention. We did in fact collect the frequency measures for each item but did not use them in the calculations in the original manuscript. Given this feedback, and reading the report that the reviewer sent, we have reconstructed our food insecurity measures at each wave incorporating the frequency variables as per the HHS protocol and have categorized children at each wave as little to no hunger, moderate hunger, and severe hunger, and have also re-calculated the food insecurity trajectories. Using this criterion, fewer children are categorized as food insecure (83.7% are never food insecure, 13.0% are intermittently food insecure, and 3.3% are chronically food insecure). The results do change slightly, but the main findings remain the same. All tables and text have been revised accordingly.

Also, given that there is now a smaller number of children in the Persistent Food Insecurity group, we have added an analysis that examines food insecurity status ever during the three-year period. We have revised our analysis to only included adjusted models, and now include the following tables / key analyses: (i) adjusted value-added models for “ever food insecure” (Table 2), and (ii) adjusted value-added models for food insecurity trajectories (Table 3).

3. Lines 147-151: Presenting background information about Ghana as a whole (e.g., rates of malnutrition, food insecurity, percentage of population below the poverty line, etc.) is a bit misleading, a the Greater Accra area, which is the setting of your analyses, is quite different from the rest of Ghana, particularly compared to the North.

**Response:** Thank you for your comment, we very much agree and have emended this section now to include a focus on the Greater Accra Region in the Introduction and Discussion sections. Specifically:

- Pages 7-8: “Notably, there is great variation across the regions across Ghana. The Greater Accra Region, in which this study takes place, is the most developed and fastest-growing region of the country, has the smallest proportion of socioeconomically disadvantaged citizens of all the regions, and is rife with ethnic diversity given rapid internal migration occurring in the region (46). Nonetheless, this study takes place in the most disadvantaged districts in the region. According to the 2014 UNICEF District League Table (a social accountability index that ranks regions and districts based on development and delivery of key basic services, including education, health, sanitation, and governance), the average ranking on “disadvantage” for the study districts ranged from 93–187 (average of 139) out of 216 districts in the country (47)”
- Page 19: “Importantly, our study took place in the Greater Accra Region of Ghana, the fastest growing and most urbanized region of the country. Importantly, the districts were some of the most disadvantaged in the region. Thus, the results should be considered in light of the disadvantaged peri-urban and urban context. Rates of food insecurity and their subsequent associations with child outcomes may differ in rural regions of the country, where poverty rates are higher and food insecurity may be closely connected to agricultural cycles and seasons (41).”

4. Line 101: Should this read “early childhood education (ECE) inputs” rather than early educational education (ECE) inputs?

**Response:** Thank you for pointing this out, we have made this correction.

5. Lines 190-196: This information should be moved to the results section (e.g., in an Attrition subsection of the Results section). And given the relatively high rate of attrition, I would like to see all of this summarized (and tested for differences by wave) in a table. Also, please include baseline food insecurity among the factors summarized and tested.

**Response:** We have moved this information to a sub-section titled “Sample Attrition,” and added in the results from the analysis comparing the differences in a table in the Online Supplementary Materials (S1). Unfortunately, we cannot compare baseline food insecurity because we have excluded children who are missing this data. Thus, we can only compare the two samples based on the data that is available for both groups.

6. Line 169: Please address how the timing of the HHS questionnaires at each of the three rounds corresponded to the agricultural season/seasonal food insecurity in Ghana.

**Response:** This is an important point, particularly in rural regions of Ghana where food insecurity may be more directly connected to the agricultural cycles. Importantly, given that this study took place in peri-urban communities in the fastest-urbanizing region of Ghana (see our response to comment #3 above), we do not think this concern is as relevant since families are much less likely to rely on subsistence farming for their food sources. The questionnaires were administered in October 2015, May 2017, and May 2018. We have included this point in the Discussion section on page 19.

7. Line 285: Equation (2) is not an equation and looks odd just hanging there. For clarity, please fully write out equation 2.

**Response:** We have made this change.

8. Line 378: Asset index? Please be consistent in your terminology.

**Response:** We have made this correction throughout and changed any reference to “wealth” or “household wealth” to “assets” or “asset index”.

9. Statistical analyses: Given the observational nature of your data, it is not clear that the unadjusted results are meaningful, as, as you mention, there are likely a number of factors that may be related to household food insecurity and child outcomes. Further, it is not clear why the household asset index, school quality, and private school control variables were not included in the “main” adjusted models, and why these three control variables were added to the model individually. Too highly correlated? To me, it seems like the most appropriate/robust estimates would be those that were generated via models that included all covariates currently included in the adjusted models plus those included in the robustness checks. Unless there is some compelling reason why the unadjusted results and the results without the household asset index and school-related covariates are needed, I would suggest presenting just the “fully adjusted” results and basing all of your discussion and conclusions on this set of results only.

**Response:** We have updated the robustness analysis to also include a fourth panel with all three covariates together. We still present the robustness models with each variable separately as they allow us to test different potential pathways through which food insecurity might operate (per the Introduction sub-section starting on page 4), alone or together. We clarified this point in the text (lines 404-407).“

10. Lines 446-447: Or that the relationship is context-specific and might vary across countries/cultures.

**Response:** We thank the reviewer for raising this, which we agree is an important point. We revised version of this sentence to add that the relationship may depend on the context, as well as highlight that this relation may also vary at different ages (page 21).

**Reviewer #3:**

11. Under statistical analysis, line numbers 280-284, the authors mentioned that they performed multivariate analyses. It will be helpful to know the type(s) of multivariate analyses performed and which software was used. Multivariate usually connotes that multiple independent variables predict multiple dependent variables in one model, whereas a multiple variable analysis connotes a dependent variable with multiple independent variables in one model. It will be helpful when the authors clearly state whether they performed a multivariate or multiple variable analysis.

**Response:** We use the term multivariate analyses to indicate that we include multiple independent variables (as opposed to bivariate) in our models. Because we immediately go on to describe the models with an equation and description, we believe this is fairly clear and straightforward for the readers. To further clarify, we have added in that software program that we used (Stata version 15.1).

12. On lines 333, 363, the authors mentioned that they obtained ‘robust confidence intervals.’ It will be helpful to know how they obtained the ‘robust confidence intervals’ and what motivates their choice of ‘robust confidence intervals.’ The concern raised here relates to the earlier one raised in the methods section.

**Response:** We obtained robust confidence intervals by clustering the standard errors at the school level. We clarified this in the notes below the tables, adding (new text in italics): “Robust confidence intervals in parentheses *based on* standard errors clustered at baseline school-level”.
