# Supplementary material for: Household food insecurity and early childhood development: Longitudinal evidence from Ghana
Source: PLoS One. 2020 Apr 3;15(4):e0230965. doi: 10.1371/journal.pone.0230965 (PMC7122750; doi:10.1371/journal.pone.0230965)
Supplement: Attachment — Submitted filename: Response to reviewer.docx [file pone.0230965.s007.docx]

We greatly appreciate the time that the editor and reviewers took to review our manuscript and provide additional feedback and comments, which improved our manuscript. We are glad that all the reviewers were satisfied with our revisions, and we respond to the additional comments made by Reviewer 2 below, thanking her/him for the helpful comments.

**Reviewer #2**

Line 166: “…and is rife with ethnic diversity…” Consider replacing the word “rife,” which often has a negative connotation, to something more neutral.

*Thanks for the suggestion. We replaced the sentence with: “*and it is characterized by considerable ethnic diversity given rapid internal migration”

Lines 163-171: Thank you for adding some additional context about the Greater Accra Region. I still think, though, that you are not completely characterizing the situation in the Greater Accra Region. Specifically, you note that (lines 149-150) “As many as 1.2 million Ghanaians are classified as food insecure and an 150 additional two million people are considered as extremely vulnerable to food insecurity (42).” However, the reference you provide for this statistic includes a map that clearly shows the Greater Accra Region categorized as the lowest percentage of people who are food insecure. Please add some information about the extent of food insecurity in the Greater Accra Region relative to the rest of the country. You could lead with this information to being the paragraph starting on line 163, and then argue, however, that your specific study districts within the Greater Accra Region are relatively disadvantaged (as you’ve done).

*Thanks for the suggestion, we moved the paragraph on Accra region just after the one in which we provide general information about Ghana, so that the comparison with the rest of Ghana is clearer.*

Equation 1 (line 324): I think Xi,3 should rather be Xi,1?

*That is correct, thank you.*

Line 365-366: Typo. “…being in a household that were ever food insecure…” Should either be household that was or households that were.

*Thanks for catching the typo, we amended it.*

Line 377: Perhaps not “interesting” but rather a result of your data. In particular, given that now only ~3% of your sample is categorized as having persistent food insecurity, the size of this “bin” is very small (perhaps too small to tease out meaningful/precisely estimated associations). You may want to note this possibility in your presentation/interpretation of these results.

*Thanks, we agree that for outcomes such as short-term memory the predictive role of persistent food insecurity might have imprecisely estimated due to small cell size. We removed “interesting” and added this point in the discussions (see lines 465-467: “*This result might be driven by small cell size in the case of persistent food insecurity, as only 3% of households were categorized as such”.)
